# Phylogenetic relationship and virulence composition of *Escherichia coli* O26:H11 cattle and human strain collections in Scotland; 2002–2020

**DOI:** 10.3389/fmicb.2023.1260422

**Published:** 2023-11-06

**Authors:** Deborah V. Hoyle, Bryan A. Wee, Kareen Macleod, Margo E. Chase-Topping, Andrew G. Bease, Sue C. Tongue, David L. Gally, Sabine Delannoy, Patrick Fach, Michael C. Pearce, George J. Gunn, Anne Holmes, Lesley Allison

**Affiliations:** ^1^Roslin Institute, Royal (Dick) School of Veterinary Studies, University of Edinburgh, Easter Bush, Edinburgh, United Kingdom; ^2^Centre for Epidemiology and Planetary Health, Department of Veterinary and Animal Science, North Faculty, Scotland’s Rural College (SRUC), Inverness, United Kingdom; ^3^Unité ColiPath – Plateforme IdentyPath, Laboratoire de Sécurité des Aliments, Agence Nationale De Sécurité Sanitaire de l’alimentation, de l’environnement et du travail (ANSES), Maisons-Alfort, France; ^4^Scottish E. coli O157/STEC Reference Laboratory (SERL), Royal Infirmary of Edinburgh, Edinburgh, United Kingdom

**Keywords:** STEC, O26:H11, cattle, phylogenetic, whole genome sequencing (WGS), virulence, epidemiology

## Abstract

O26 is the commonest non-O157 Shiga toxin (*stx*)-producing *Escherichia coli* serogroup reported in human infections worldwide. Ruminants, particularly cattle, are the primary reservoir source for human infection. In this study, we compared the whole genomes and virulence profiles of O26:H11 strains (*n* = 99) isolated from Scottish cattle with strains from human infections (*n* = 96) held by the Scottish *Escherichia coli* O157/STEC Reference Laboratory, isolated between 2002 and 2020. Bovine strains were from two national cross-sectional cattle surveys conducted between 2002–2004 and 2014–2015. A maximum likelihood phylogeny was constructed from a core-genome alignment with the O26:H11 strain 11368 reference genome. Genomes were screened against a panel of 2,710 virulence genes using the Virulence Finder Database. All *stx*-positive bovine O26:H11 strains belonged to the ST21 lineage and were grouped into three main clades. Bovine and human source strains were interspersed, and the *stx* subtype was relatively clade-specific. Highly pathogenic *stx*2a-only ST21 strains were identified in two herds sampled in the second cattle survey and in human clinical infections from 2010 onwards. The closest pairwise distance was 9 single-nucleotide polymorphisms (SNPs) between Scottish bovine and human strains and 69 SNPs between the two cattle surveys. Bovine O26:H11 was compared to public EnteroBase ST29 complex genomes and found to have the greatest commonality with O26:H11 strains from the rest of the UK, followed by France, Italy, and Belgium. Virulence profiles of *stx*-positive bovine and human strains were similar but more conserved for the *stx2a* subtype. O26:H11 *stx*-negative ST29 (*n* = 17) and ST396 strains (*n* = 5) were isolated from 19 cattle herds; all were *eae*-positive, and 10 of these herds yielded strains positive for *ehxA*, *espK*, and *Z2098*, gene markers suggestive of enterohaemorrhagic potential. There was a significant association (*p* < 0.001) between nucleotide sequence percent identity and *stx* status for the bacteriophage insertion site genes *yecE* for *stx2* and *yehV* for *stx1*. Acquired antimicrobial resistance genes were identified *in silico* in 12.1% of bovine and 17.7% of human O26:H11 strains, with s*ul2*, *tet*, *aph(3″),* and *aph(6″)* being most common. This study describes the diversity among Scottish bovine O26:H11 strains and investigates their relationship to human STEC infections.

## 1. Introduction

Shiga toxin-producing *Escherichia coli* (STEC) are a group of zoonotic pathogenic bacteria with a ruminant reservoir that cause gastrointestinal infections in humans (Kolenda et al., 2015; World Health Organization and Food and Agriculture Organization of the United Nations, 2018). Transmission to humans occurs via foodborne routes, as well as by direct contact with infected animals and through environmental contamination, particularly water (Kintz et al., 2017). Shiga toxin is the primary virulence factor responsible for severe pathology and is encoded by *stx* genes hosted on mobile lysogenic bacteriophage, which integrate into the bacterial genome at specific insertion sites (Bonanno et al., 2015). There are two main Shiga toxin proteins, namely, Stx1 and Stx2, encoded by different gene subtypes *stx1a, 1c, 1d, 1e,* and *stx2a-o* (Scheutz et al., 2012; Hughes et al., 2019; Gill et al., 2022), with the *stx2a*, *2c*, and *2d* subtypes associated with more serious disease (Friedrich et al., 2002; Persson et al., 2007). The majority of STEC are typically characterised by the presence of the locus of enterocyte effacement (LEE), which is required for the formation of attaching and effacing lesions in the intestine and encodes the intimin gene, *eae*, in addition to a number of other key virulence factors (Kaper et al., 2004). However, LEE is not essential for human pathogenicity, with some LEE-negative non-O157 STEC serotypes still capable of causing severe disease, mediated by other virulence determinants (Newton et al., 2009; Colello et al., 2019). The LEE is not specific to the STEC pathotype and is also found in the majority of enteropathogenic *E. coli* (EPEC), which cause non-haemorrhagic gastrointestinal illness in both animals and humans; EPEC are primarily distinguished from STEC by the absence of the *stx* gene (Denamur et al., 2021).

STEC cause a spectrum of clinical symptoms in humans, from uncomplicated diarrhoea to haemorrhagic enteritis, haemolytic uraemic syndrome (HUS), and, in exceptional cases, death. STEC serotypes that are responsible for the more severe, haemorrhagic disease presentations are further classified as enterohaemorrhagic (EHEC) (Nataro and Kaper, 1998). Globally, *E. coli* O157:H7 is the commonest STEC/ EHEC serotype and is often associated with large foodborne outbreaks of disease. However, a number of non-O157 STEC serotypes can also be classified as EHEC based on their disease and pathogenicity profile, with O26:H11 being the predominant non-O157 serotype of clinical relevance in human cases worldwide (Caprioli et al., 2005; Johnson et al., 2006). In Europe, O26:H11 frequently surpasses O157:H7 reported cases and is currently the leading serotype responsible for human STEC infection, including paediatric HUS (European Centre for Disease Prevention and Control, 2022).

The recent increase in the proportion of STEC clinical cases attributed to non-O157 serotypes may in part be due to improvements in diagnostic testing methods (Parsons et al., 2016). However, the emergence of two highly pathogenic, *stx2*-only positive O26:H11 clones, termed the new “European” and “French” clones, has also resulted in a true increase in O26:H11 incidence across Europe over the past decade (Bielaszewska et al., 2013; Zweifel et al., 2013; Delannoy et al., 2015). These clones have been particularly associated with disease outbreaks and hospitalisations in children, linked to the consumption of dairy products in France, Italy, and Romania (Severi et al., 2016; Jones et al., 2019; Loconsole et al., 2020).

Globally, O26:H11 strains can be grouped into two main multilocus sequence types (MLSTs), namely, ST21 and ST29 (Ogura et al., 2017). The majority of all *stx*-positive O26:H11 strains belong to ST21, which includes the predominant *stx1*-only strains as well as dual positive *stx1* + *stx2* strains and less common strains encoding *stx2a* only. ST29 comprises mostly *stx*-negative O26:H11 strains but also includes the newly emerging *stx2a* + “European” and *stx2d* + “French” clones. STEC harbour large virulence plasmids (pVF) that host genes for enterohaemolysin, *ehxA*, catalase-peroxidase, *katP*, serine protease, *espP*, and a type II effector protein, *etpD* (Fratamico et al., 2011; Bielaszewska et al., 2013). The main O26:H11 lineages are distinguished by the presence or absence of these pVF genes, with the ST21 lineage characterised by the *ehxA*+/*katP*+/*espP*+/*etpD*- gene profile. In contrast, the newly emerging and highly virulent ST29 European *stx2*+ clone bears a distinct *ehxA*+/*katP*-/*espP*-/*etpD*+ pVF gene profile (Bielaszewska et al., 2013; Ogura et al., 2017).

ST29 *stx*-negative O26:H11 strains that carry the *eae* gene are classed as EPEC; however, a subset of these strains has also been shown to carry the *ehxA*+/*katP*+/*espP*+/*etpD*- pVF gene profile typically seen in *stx*-positive ST21 strains, together with a range of additional virulence factors (Leomil et al., 2005). Such *stx*-negative strains have been termed “EHEC-like” because the acquisition of the *stx* gene through bacteriophage lysogeny could result in conversion to a highly virulent EHEC pathogenic strain profile (Bielaszewska et al., 2007; Bugarel et al., 2011). To distinguish between O26:H11 *stx*-negative EPEC and EHEC-like strains, an additional set of genetic markers has been proposed to assist in the identification of strains with EHEC potential (Bugarel et al., 2011; Delannoy et al., 2013a). These markers include the type III secretion system genes *espK* (Vlisidou et al., 2006), urease gene *ureD* (Steyert et al., 2011), and the open reading frame putative marker Z2098 (Delannoy et al., 2013b).

Scotland has a higher incidence of human STEC infections than the EU average and has reported an increased incidence of non-O157 serotypes isolated from clinical patients in recent years (Food Standards Scotland, 2020; Public Health Scotland, 2020). To assess the prevalence and distribution of STEC in Scottish cattle, two national cross-sectional surveys were conducted in 2002–2004 (Pearce et al., 2006, 2009) and 2014–2015 (Henry et al., 2017), from which a collection of bovine-sourced *stx*-positive and negative O26 *E. coli* strains were isolated (Pearce et al., 2006; Hoyle et al., 2021). The aim of this present study was to compare by whole-genome sequencing the O26:H11 strains isolated from bovine faecal samples collected through these two Scottish cattle surveys with clinical O26:H11 human strains isolated from patients and previously sequenced by the Scottish *E. coli* O157/STEC Reference Laboratory (SERL) (Food Standards Scotland, 2020). We also further examined how the Scottish bovine strains related to human-derived O26:H11 strains from across the wider UK (Dallman et al., 2021; Rodwell et al., 2023) and investigated their global O26:H11 phylogenetic context by comparison with the public collection of clonal complex 29 genomes deposited within EnteroBase (Zhou et al., 2020).

Ongoing analysis of strains from reservoir hosts such as cattle is essential for monitoring the microevolution and emergence of new pathogenic STEC and EHEC strains. These data inform on the risk and can assist in the public health management of this pathogen.

## 2. Materials and methods

### 2.1. Bacterial genomes included in the study

In total, 195 O26:H11 *E. coli* strains from Scottish cattle (*n* = 99) and the Scottish human strain collection (*n* = 96) were included in the analysis, together with 3 O177:H11 bovine strains that fell within the ST29 complex and a single bovine O103:H14 strain as an outgroup (Table 1; Supplementary Table 1).

**Table 1:** Summary of the bovine and human bacterial strain genomes included in this study.

Strain collection (Year)	Serotype	*stx* gene profile	Number of genomes	References
Archive cattle (2002–2004)	O26:H11	*stx1* *stx1* + *stx2* *stx*-negative	41 16 3	Pearce et al. (2006)
BECS cattle (2014–2015)	O26:H11	*stx1* *stx2* *stx1* + *stx2* *stx*-negative	10 4 6 19	Hoyle et al. (2021)
	O177:H11	*stx1* *stx*-negative	2 1	
	O103:H14	*stx*-negative	1	
Human (SERL) (2002–2020)	O26:H11	*stx1* *stx2* *stx1* + *stx2*	56 6 34	Food Standards Scotland (2020)

Bovine strains were originally isolated from cattle faecal pat samples that had been obtained during two cross-sectional surveys of Scottish cattle farms conducted between 2002–2004 (Pearce et al., 2006) and 2014–2015 (Henry et al., 2017; Hoyle et al., 2021), as previously described. In both surveys, the original faecal pat samples were collected by sampling discrete, dropped, faecal pats present on the ground of grazing land or the floor of pens.

The cattle strain collection comprised 60 isolates obtained from 35 herds, sampled in the 2002–2004 survey (Archive), and 43 isolates obtained from 29 herds in the 2014–2015 study (BECS). In the initial survey, Scotland was divided into six distinct geographical animal health district regions, as previously outlined (Pearce et al., 2006), and herds were therefore also grouped according to this geographic classification in the second survey.

Human clinical O26:H11 genomes were provided from a collection of genome sequences held by the SERL. Clinical O26:H11 human strains were originally isolated from faecal sample submissions received by the SERL between 2002 and 2020 that were PCR-positive for *stx* genes. A subset of the human genomes sequenced at the SERL was included in this study, selected as described below in phylogenetic analysis (2.3). Only a single representative genome from any outbreak-linked human strain was included in this comparative analysis.

Detailed methods used by the SERL for extraction, PCR, library preparation, sequencing, and analysis have been described elsewhere (Food Standards Scotland, 2020). In brief, genomic DNA was extracted either manually with the DNeasy Blood and Tissue Kit (Qiagen, Crawley, UK) or with the QIAsymphony using the QIA DSP DNA Mini Kit (Qiagen). Libraries were prepared using the Nextera XT kit and sequenced on the Illumina MiSeq, producing paired-end reads of 250 bp. Sequencing reads were processed in BioNumerics using the wgMLST and *E. coli* genotyping plug-in tools. The assembly was performed using SPAdes, and basic assembly metrics were calculated for quality assessment. Sequencing reads were also processed using the Scottish Microbiology Reference Laboratory Edinburgh Bioinformatics Pipeline (SMiRLWBP). Trimmomatic (Bolger et al., 2014) was used to remove bases with a Phred score < 30 from the trailing edge. KmerID (Chattaway et al., 2017) identified bacteria species, and the GeneFinder tool mapped reads to a panel of serotype and virulence genes using Bowtie2 (Langmead et al., 2009). Only *in silico* predictions of serotype and virulence that matched a gene determinant at >80% nucleotide identity and over >80% target gene length were accepted. MLST alleles of seven housekeeping genes (*adk*, *fumC*, *gyrB*, *icd*, *mdh*, *purA*, and *recA*) were determined using the Metric-Oriented Sequence Typer (MOST) (Tewolde et al., 2016). Shiga toxin gene subtyping was performed using a combined mapping and BLAST approach as previously described (Ashton et al., 2015).

The funding bodies approved and authorised informed consent documentation for farm survey participants to enable the collection of dropped faecal pat samples from participant land. Permission had been granted and consent obtained for the samples, strains, and data to be used for further research. All farm participants’ personal data were handled in accordance with the UK Data Protection Act (1998) and are now handled in accordance with the UK General Data Protection Regulation (GDPR 2018).

The de-identified bacterial genomes from human clinical samples were obtained from the National Health Service (NHS) Lothian following ethics approval from the biorepository bank in NHS Lothian covering the sequencing of samples (20/ES/0061).

### 2.2. DNA extraction and sequencing of cattle-sourced bacterial strains

DNA was extracted from the cattle-sourced bacterial strains using the DNeasy Blood and Tissue Kit (Qiagen, Crawley, UK). The quantity of DNA was measured using the Qubit Fluorimeter 3.0 (Thermo Fisher Scientific) with the dsDNA Assay HS Kit. The *stx* subtype for all bovine isolates was determined initially by PCR as previously described (Pearce et al., 2006; Hoyle et al., 2021), and library preparation, sequencing and analysis for serotype, MLST, and Shiga toxin subtyping were performed at the SERL, as described above.

### 2.3. Phylogenetic analysis

A subset of the Scottish human strain genomes held by the SERL that differed by fewer than 50 cgMLST (core-genome MLST) alleles from bovine strains was determined using an *ad-hoc* cgMLST schema with chewBBACA v2.5.5 (Silva et al., 2018), using allele profiles downloaded from EnteroBase^1^ and made available for comparison with the bovine genomes. This subset of human strain genomes was provided as paired Illumina raw reads for full comparative analysis with all bovine strain genomes. Raw reads were quality filtered using bbduk (v38.45) with the settings ‘k = 19 mink = 11 hdist = 1 ktrim = r minoverlap = 12 qtrim = rl trimq = 20 minlength = 50’ (Bushnell, 2019). All genomes were assembled using SPAdes (v3.15.3) with the --careful option (Bankevich et al., 2012). Criteria for inclusion were 50–51% GC, a total assembly length of 4.5–6.5 Mbp, a duplication ratio below 1.021, and a minimum N50 of 40 Kbp. Genome alignment was performed using Parsnp v1.5.6 with reference genome AP010953.1 (O26:H11 strain 11368) (4.03 Mbp, 35,612 polymorphic sites) (Treangen et al., 2014). Recombinant SNPs were filtered using Gubbins with default settings (v3.0.0) (Croucher et al., 2015). A maximum likelihood tree (IQTree v2.1.2) was constructed from the filtered core-genome alignment of 198 sequences with the TVMe+ASC model and 1,000 bootstraps (Minh et al., 2020). Figures were generated using iTol (Letunic and Bork, 2021).

### 2.4. Virulence, phage insertion site, and antimicrobial resistance gene identification

All genomes were screened against a panel of 2,710 virulence genes from the *E. coli*-specific virulence gene database (Date downloaded: 10 September 2021) using Abricate (v1.0.1), supplemented with 139 additional gene alleles of interest, run with default parameters (−-minid 80, −-mincov 80) (Supplementary Table 2)^2^ (Escherichia coli Virulence Factors, 2021; Seemann, 2022a). The percent identity was recorded, and the gene target was categorised as positive or negative based on whether the gene was detected using Abricate threshold parameters of minimum 80% coverage and 80% identity. Additional targets included the *espK*, *ureD*, and *Z2098* genes, which were thought to be indicative of the potential pathogenicity of O26:H11 *stx*-negative strains following the acquisition of the *stx* gene (Bugarel et al., 2011; Delannoy et al., 2013a). Sequences for these genes were sourced from the O26:H11 reference strain 11368, GenBank accession number AP010953. Genomes were additionally screened using Abricate for the presence of four phage insertion site genes commonly associated with *stx1* and *stx2* bacteriophage insertion into the O26 *E. coli* serogroup: *yecE* and *wrbA* for *stx2* and *yehV* and *sbcB* for *stx1* (Bonanno et al., 2015).

To examine the potential for antimicrobial resistance, strain genomes were screened for acquired antimicrobial resistance genes (ARGs) using StarAMR (v0.5.1), and the ResFinder gene database (downloaded 7 September 2021) using default parameters (--pid-threshold 98, --percent-length-overlap 60) (Bharat et al., 2022; Florensa et al., 2022). Cattle strains positive for ARGs were submitted to the web-based Mobile Genetic Element Finder^3^ on 17–30 October 2022, to examine whether identified ARGs were associated with particular mobile genetic elements (Johansson et al., 2021).

The local arrangement of genes within the genome was examined for the single integron-bearing bovine strain and for the exploration of bacteriophage insertion site genes in bovine strains using Artemis 18.1.0 (Carver et al., 2012). Insertion site genes were identified using the navigator tool with primer sequences, according to Bonanno et al. (2015). Nucleotide and amino acid sequences were extracted as FASTA and verified in BLAST^4^ and UniProt^5^ (Camacho et al., 2009; The UniProt Consortium, 2021).

The potential carriage of any prophage sequences was assessed for all bovine *stx*-negative strains by submission to PHASTER^6^ in March/November 2022 (Arndt et al., 2016).

### 2.5. Comparison with publicly available worldwide genomes for *Escherichia coli* ST29 complex

Publicly available genomes from the *E. coli* ST29 complex (*n* = 8,511; Supplementary Table 3) were downloaded from EnteroBase on 16 March 2022 (Zhou et al., 2020). The HeirCC HC-1100 cluster “2” was used to filter genome assemblies, and we further selected genomes for which “Country” metadata were available (*n* = 8,332, from 36 countries).

### 2.6. Pairwise distance in core-genome alignment between Scottish bovine, human, and publicly available O26:H11 genomes

A core-genome alignment of the downloaded EnteroBase genomes in the *E. coli* ST29 complex, together with 198 of the genomes from this study, was generated using Snippy (v4.6.0) against the O26:H11 strain 11368 genome (AP010953.1), and the pairwise distances between genomes were calculated using Disty (v0.1.0) (Disty McMatrixface, 2021; Seemann, 2022b). A conservative threshold of 200 core SNPs was used to capture clusters of epidemiologically linked isolates (Dallman et al., 2015). Distances were compared within herds, between herds, and between cattle and humans within Scotland, as well as between cattle and non-UK country O26:H11 genomes.

### 2.7. Statistical analysis

Fisher–Freeman–Halton exact tests for comparing category proportions were performed in StatXact Version 11 (Cytel Inc., Cambridge, MA, US). Associations between binary virulence gene occurrence, *stx* profile, and host species for all *stx*-positive O26:H11 genomes were performed using non-metric multidimensional scaling (NMS), PC-ORD software version 7.04 (MJM Software Design, Gleneden Beach, OR, US). Strains that were *stx*-negative (*n* = 22) were excluded from the analysis since the almost complete separation in virulence profiles between ST396 and the two differing clusters of ST29 prevented the model from reaching a stable solution. Genes were excluded from the analysis where they were present in <4% or > 96% of the samples, identical to or highly correlated with other genes (Supplementary Table 4; Supplementary Figure 1). The final NMS was run with 30 genes and 173 strain genomes. NMS was used with a grower distance measure. The dimensionality of the dataset was determined by plotting an inverse measure of fit (“stress”) to the number of dimensions. Optimal dimensionality was based on the number of dimensions with the lowest stress. A three-dimensional solution was shown to be optimal. Several NMS runs were performed for each analysis to ensure that the solution was stable and represented a configuration with the best possible fit. On this basis, 500 iterations were used for each NMS run, using random starting coordinates.

## 3. Results and discussion

### 3.1. Phylogenetic analysis of O26:H11 genomes

**Figure 1:**
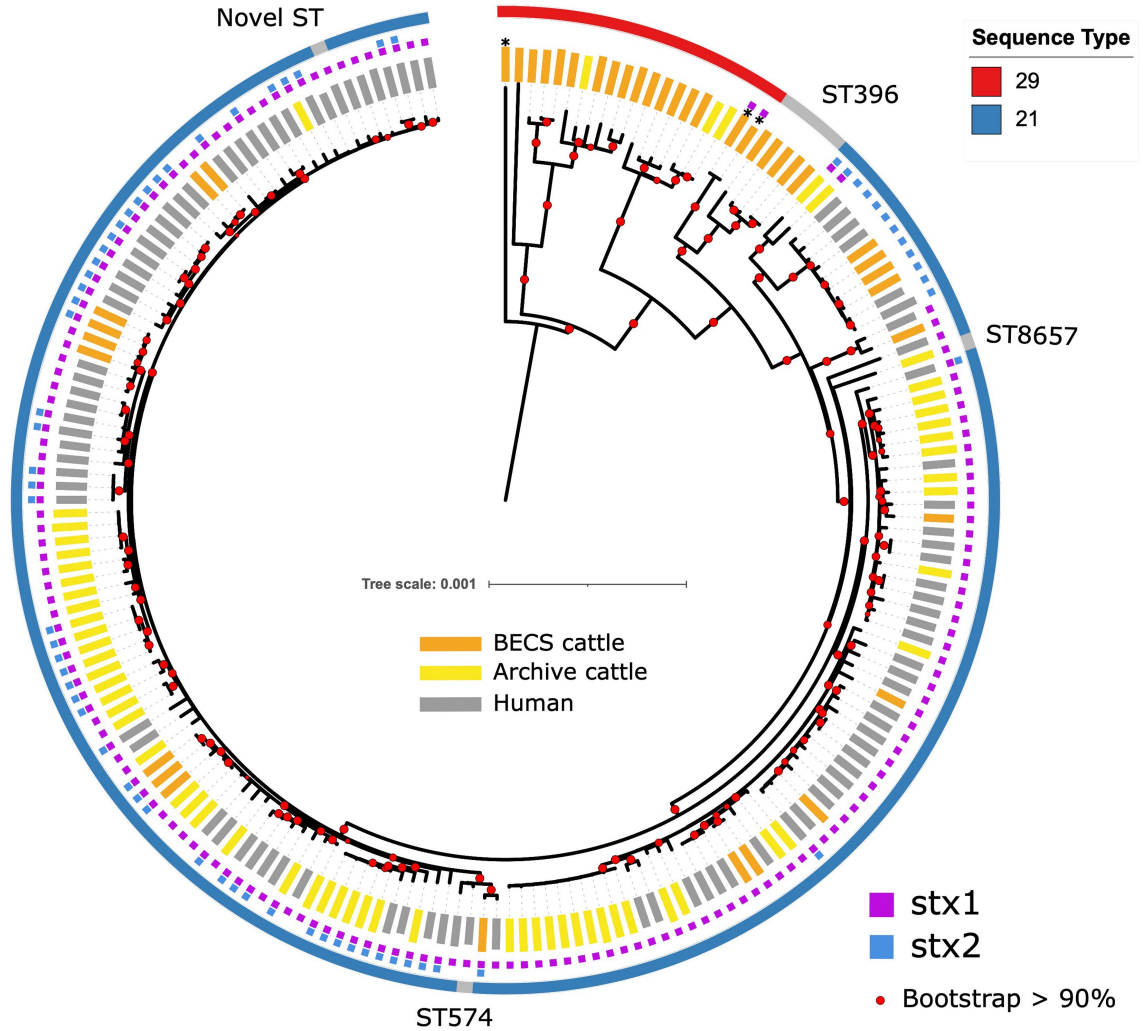
Maximum likelihood core-genome phylogenetic analysis of Scottish bovine and human O26:H11 strains (*n* = 195) and three O177:H11 strains (*) analysed in this study. The inner ring shows the presence of the *stx1* and *stx2* genes and the outer ring shows the MLST number. The tips are coloured by source and dataset [cattle – BECS (orange) or Archive (yellow); human clinical (grey)]. Red circles indicate branches with >90% bootstrap support. Tree scale is in substitutions per site.

All O26:H11 strains belonged to one of the three sequence types: ST21 (87.2%, 170/195), ST29 (8.7%, 17/195), and ST396 (2.6%, 5/195), or other single-locus variants of these STs (*n* = 3) (Figure 1). All *stx*-positive O26:H11 bovine strains belonged to ST21, as did the majority of the human strains included in this analysis, whilst all *stx*-negative O26:H11 bovine strains belonged to either the ST29 or ST396 lineages. Three bovine O177:H11 strains that were also typed as ST29 were included in the phylogeny: two *stx1a*-positive strains from a single herd, which also yielded a *stx*-negative O26:H11 strain, and one *stx*-negative strain from a herd yielding *stx2a* + *stx1a*-positive O26:H11 strains. This ST distribution of O26:H11 strains in Scotland is highly similar to that reported for human cases in England (Dallman et al., 2021) and broadly in line with the most recent phylogenetic analysis of worldwide O26:H11 genomes by Long et al. (2022), in which 84 and 16% of strains were identified as ST21 and ST29, respectively.

Within the ST21 lineage, bovine and human *stx*-positive strains were interspersed within a relatively diverse phylogeny, comprising three main clades (Figure 1). The first clade contained *stx2a*-only positive O26:H11 cattle and human strains, which falls within the ST21C1a sub-lineage, as described by Ogura et al. (2017). The second clade comprised predominantly *stx1a*-positive strains only, with two exceptions in human clinical strains, where *stx2a* was also present. The third clade contained a combination of both *stx1*-only and dual *stx2* + *stx*1-positive strains. Strain ST and *stx* subtypes are outlined in Supplementary Table 1.

The first *stx2a*-only human strains isolated from clinical samples in Scotland were recorded in 2010 (Food Standards Scotland, 2020). We did not identify any *stx2a*-only positive bovine strains in the 2002–2004 cattle survey (*n* = 338 sampled herds); however, we isolated *stx2a*-only positive strains from two herds in the 2014–2015 survey (*n* = 110 sampled herds) that clustered with ST21 human *stx2a*-only strains (Table 1; Supplementary Table 1). This *stx2a*-only lineage has not been reported in Scottish cattle prior to this survey and is most likely explained by a relatively recent introduction into Scottish cattle after the estimated emergence of this clade in the mid-20th century (Ogura et al., 2017).

We did not identify any Scottish *stx*-positive bovine strains belonging to the newly emerging ST29, highly pathogenic *stx2a*-only new European clones (Bielaszewska et al., 2013; Karnisova et al., 2018) which have been isolated from clinical cases throughout Europe and at low levels in Japan (Ishijima et al., 2017). ST29 *stx2a*-only strains have been isolated from <3% of Scottish clinical O26:H11 infections (Food Standards Scotland, 2020). However, these strains were above the 50 cgMLST genetic distance threshold to Scottish bovine strains in our initial cgMLST-based screening, the limit for the selection of clinical strains for inclusion in the comparative phylogeny. Metadata reported for clinical submissions to the national reference laboratory in England indicated that ST29 *stx*2a-positive strains isolated from human cases reported within the UK were predominantly associated with travel abroad (Dallman et al., 2021). These data would suggest that if ST29 *stx*2a-only O26:H11 strains are present and circulating within the UK cattle population, this is not currently resulting in identified human infection.

We were interested in investigating the potential for O26:H11 pathogenicity in Scottish cattle regardless of *stx* status and therefore sequenced strains from each of the 19 herds yielding *stx*-negative isolates (*n* = 22). The majority of *stx*-negative strains belonged to ST29 (*n* = 17 strains), although four herds yielded minority strains (*n* = 5) in ST396, a single-locus variant of ST29 (Bielaszewska et al., 2013; Figure 1). The ST29 genomes were split into two distinct clades: a relatively conserved clade phylogenetically closer to the ST21 lineage and a more diverse clade at a greater distance. The separation of *stx*-negative ST29 into distinct lineages, with one sub-lineage phylogenetically closer to *stx*-positive ST21, is a broadly similar finding to that reported by Ogura et al. (2017). This also concurs with the population structure observed in ST29 *stx*-negative O26:H11 strains isolated from both the US and New Zealand cattle populations (Gonzalez-Escalona et al., 2016; Browne et al., 2018) and with the recent clade classification of *stx*-negative ST29 lineages by Long et al. (2022). Three Archive bovine strains that had been confirmed as *stx*-positive by PCR at the SERL prior to long-term cryostorage were found to be *stx*-negative on resuscitation, by both genome sequencing and repeat PCR. This may have been due to the spontaneous excision of the *stx*-encoding prophage.

### 3.2. Pairwise distances between bovine isolates from the two Scottish cattle surveys

**Figure 2:**
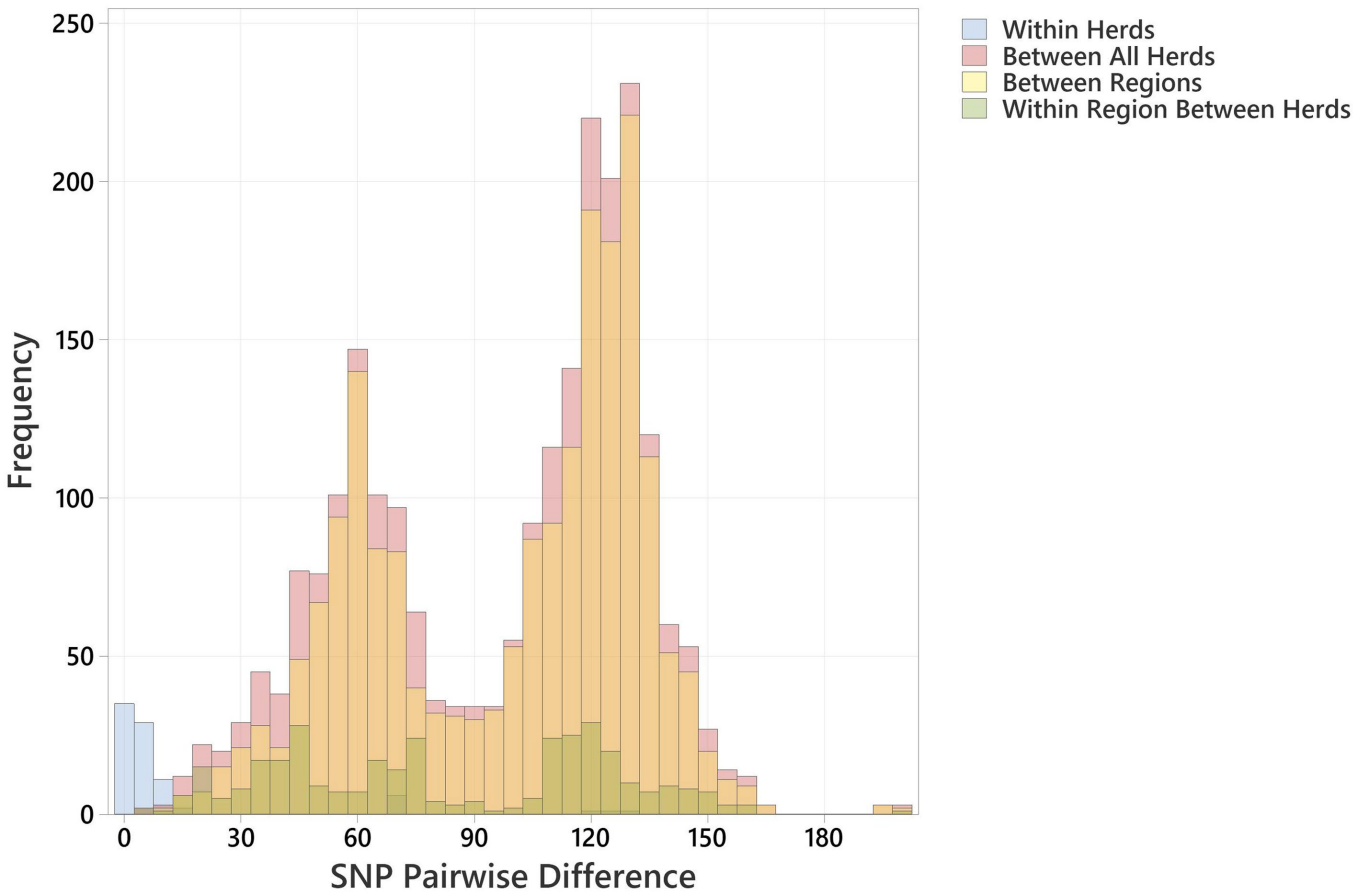
Histogram showing pairwise SNP differences <200 SNP, between bovine O26:H11 strains (*n* = 99), for the comparisons “Within Herd,” “Within Region” (excludes Within Herd), “Between Regions” and “Between All Herds.”

Multiple O26:H11 isolates from individual herds were available for 10 Archive and 7 BECS herds, and 47 herds yielded a single strain per herd (Supplementary Table 1). Multiple strains within herds were compared to examine within-herd diversity. Overall, the closest observed relationship was within-herd/within-survey, with a minimum pairwise difference of 0 SNP and median of 3 (interquartile range, IQR = 5) (Figure 2; Supplementary Table 5), although strains within-herd from the BECS survey had closer SNP distances than those within-herd from the Archive survey. Ten *stx1a* genomes were available for a single Archive herd and gave a median pairwise difference of 4 SNPs and a maximum of 15 SNPs, clustered within a single node. These data suggest that within the study, O26:H11 isolates with the same *stx* profile spread clonally at the herd level, rather than supporting multiple lineage introductions across a herd cohort. A similar observation has been reported for O26:H11 within cattle herds in New Zealand (Browne et al., 2018). However, where the *stx* profile differed within a herd, the SNP difference was found to be 128 SNPs or more, reflecting the presence of different circulating lineages. Two herds yielded strains with the same *stx* subtype in both surveys; however, the genetic distance between these isolates was 69 SNPs or more. This also suggested the presence of distinct lineages, since this pairwise distance is higher than the expected variation from a single lineage over a 10-year period (Dallman et al., 2021).

Regionally, across both cattle surveys, the median SNP difference between genomes from different herds within an animal health district was 76 (IQR 77), and between genomes across different animal health districts, the median SNP difference was 112 (IQR 63) (Figure 2; Supplementary Table 5). These data indicate that herds that were geographically closer showed closer genetic relationships between strains. This is contrary to observations on regional differences reported for New Zealand (Browne et al., 2018) and may be due to stock movements being more limited by geographical distance within Scotland, particularly for herds based on Scottish islands.

### 3.3. Pairwise distances between Scottish bovine isolates, Scottish human isolates, and other closely related O26:H11 genomes around the UK and globally

A total of 3,969 (47.6%) publicly available genomes within the HierCC:1100 (core-genome ST complex) clade 2 from EnteroBase were found to be within 200 core SNPs from Scottish bovine strain genomes (Supplementary Tables 3, 6). These genomes represented 24 countries; countries excluded that fell beyond the 200 SNP threshold were located in South America, Africa, or Asia, and yielded fewer than five genomes each, except for China, for which 11 genomes were available (Supplementary Table 6).

**Figure 3:**
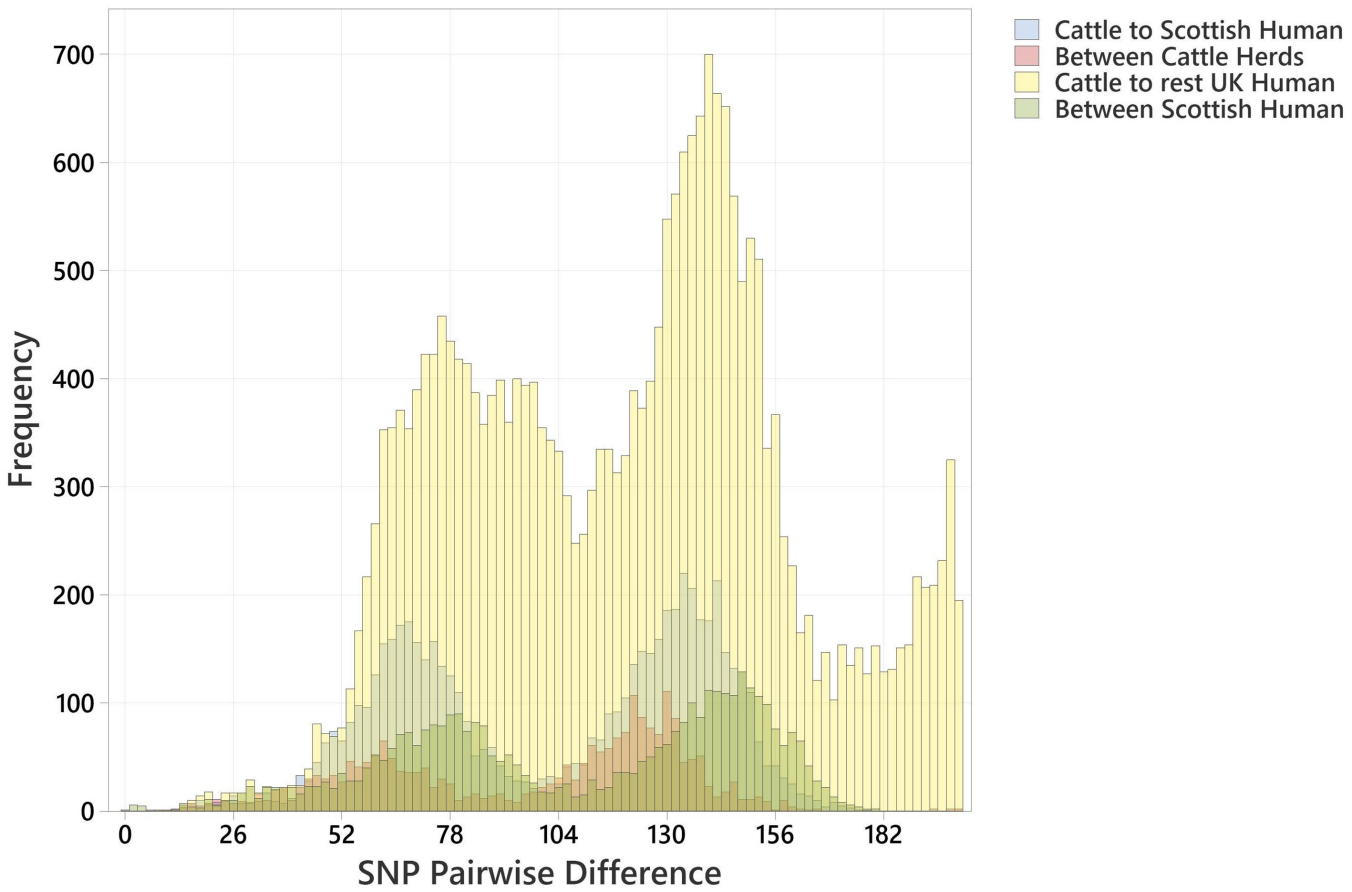
Histogram showing pairwise SNP differences between O26:H11 bovine strains (*n* = 99), Scottish human strains (*n* = 96), and rest of UK human strains (*n* = 1,217), displaying comparison with <200 SNP difference. Comparisons are given as “Cattle to Scottish Human,” “Between Scottish Cattle Herds,” “Cattle to rest UK Human,” and “Between Scottish Human.” Pairwise comparisons include bovine strains across both surveys.

The distribution profiles for pairwise SNP differences between bovine strains, between human strains, and between cattle and humans within Scotland and across the rest of the UK were very similar (Figure 3). All showed a biphasic distribution, due to the presence of two major clusters of strains, with a similar median pairwise SNP difference in Scotland of cattle to cattle (between herds), cattle to human, and human to human of 110 (IQR 63), 114 (IQR 66), and 118 (IQR 70), respectively (Supplementary Table 5). The closest relationship between any bovine strain and a Scottish human strain was 9 SNPs between a BECS isolate (2014) and a human strain isolated in 2019. The closest relationship to any UK human strain was 7 SNPs between this same BECS strain and a non-Scottish UK human strain isolated from an individual with diarrhoea from the South of England in 2015 (Supplementary Table 6). For the latter example, the close relationship between the South England human strain and the Scottish cattle strain could be due to either the movement of cattle or of a bovine-contaminated food source from Scotland to South England or through human travel to and consequent infection within Scotland. This study does not allow us to draw any conclusions on directionality or source attribution; however, these data could indicate a common source reservoir for the majority of ST21C1b lineage strains found in cattle and humans within the UK.

**Figure 4:**
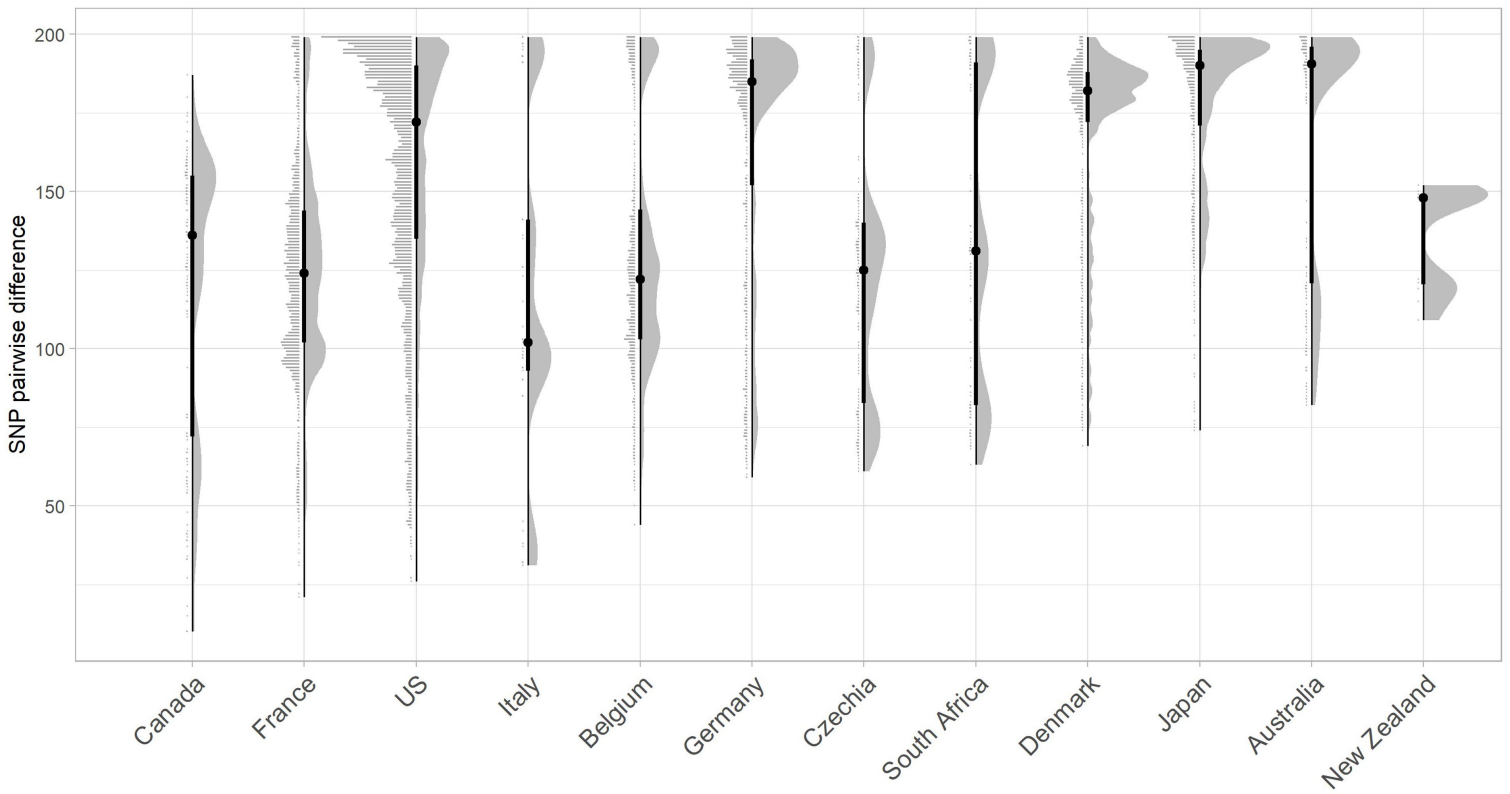
Raincloud plot for the pairwise SNP difference between bovine O26:H11 strain genomes (*n* = 99) to publicly available O26:H11 genomes from other countries present within the EnteroBase *E. coli* ST29 complex. Pairwise comparisons were displayed for bovine strains across both surveys to countries where five or more genomes match bovine strains <200 SNPs. Error bars illustrate the median and interquartile range.

The closest pairwise relationship observed between a Scottish bovine strain and an external country genome was 10 SNP between three bovine strains from Archive_24 and a Canadian strain (ESC_IB7316AA_AS), followed by 21 SNP between a 2019 French strain (ESC_FB8524AA_AS) and a BECS_5 strain, and 26 SNP between an Archive_14 strain and a 2018 isolate from the United States (ESC_RA4669AA_AS) (Figure 4; Supplementary Tables 3, 6). The strain source for all these closest genomes was designated as “Human” origin in the EnteroBase “Source Type” field. The 10 SNP difference between the Archive bovine strains and the human-sourced Canadian strain is unexpectedly close. The metadata associated with this Canadian strain indicate source as a human with gastroenteritis but do not provide information on the isolation date. The second-closest Canadian strain at 33 SNPs to a BECS bovine strain is attributed to the Canadian Food Inspection Agency. The nearest pairwise difference to a designated bovine source genome in the Canadian dataset was 134 SNPs in a Canadian bovine faecal sample collected in 2014.

Whilst two of the three non-UK strains that were closest matching to Scottish bovine strains were of North American origin, overall, considering the proportion of genomes matched within 200 SNPs, together with pairwise differences, Scottish cattle most closely matched strains from Europe than elsewhere, with the greatest commonality seen with France and Belgium (Figure 4; Supplementary Table 6). More than 60% of downloaded genomes from France, Italy, Belgium, and Germany were within 200 SNPs of our Scottish bovine strains, compared with less than 40% for North American strains. Japan was an unusual outlier, matching 80% of genomes within 200 SNPs of Scottish cattle. It has been previously noted that Japan imported relatively high levels of cattle from Western countries during the second half of the 20th century (Browne et al., 2019), which may account for the closer relationship of Scottish bovine strains to O26:H11 strains from Japan than to other Asian-Pacific countries. An analysis of source type by country was not performed due to a lack of available metadata within EnteroBase for the majority of the downloaded genomes.

Defining a core-genome alignment depends on the diversity and quality of the genomes included in the analysis. In this study, for the initial screen to identify Scottish human O26:H11 genomes that clustered with the bovine O26:H11 genomes, we used a reference-free cgMLST clustering approach, which would be less affected by sequence quality. For the subsequent in-depth analyses, we used the more conservative Parsnp whole-genome aligner to align the Scottish human and bovine genomes. For all comparisons of pairwise distances, we used the more robust short sequence mapping-based Snippy-Core-SNP approach, taken from the same core-genome alignment generated using Snippy-Core. The size of the alignment was approximately 276,190 SNPs. The pairwise distances calculated here are only meaningful when used to compare subsets of genomes that were included in the same core-genome alignment. This is because the core genome, by definition, is the collection of nucleotide positions that are conserved across all the genomes in the given alignment and can change according to the diversity of genomes being included.

### 3.4. Virulence gene profiles

All 195 O26:H11 genomes were screened against an *E. coli-*specific virulence gene database, together with selected additional gene targets (Escherichia coli Virulence Factors, 2021), and recorded as positive or negative according to the described Abricate threshold parameters (Supplementary Tables 4, 7). In total, a conserved set of 154 genes was identified as present in all genomes irrespective of *stx* status, including the key virulence factor genes *eae*, *tir*, *cif*, *espA*, and *espB*, encoded on the LEE pathogenicity island. Other common virulence genes present across all strains included *fim D, fim F-H*, *gadX*, *iss*, and *lpfA*, and the non-locus of enterocyte effacement effector (*nle*) genes *nleB1*, *nleG7*, *nleG8,* and *nleH1*. This observation concurs with the typical O26:H11 virulence profiles previously reported in bovine EPEC O26:H11 strains from the United States and in STEC O26:H11 worldwide (Gonzalez-Escalona et al., 2016; Long et al., 2022).

**Figure 5:**
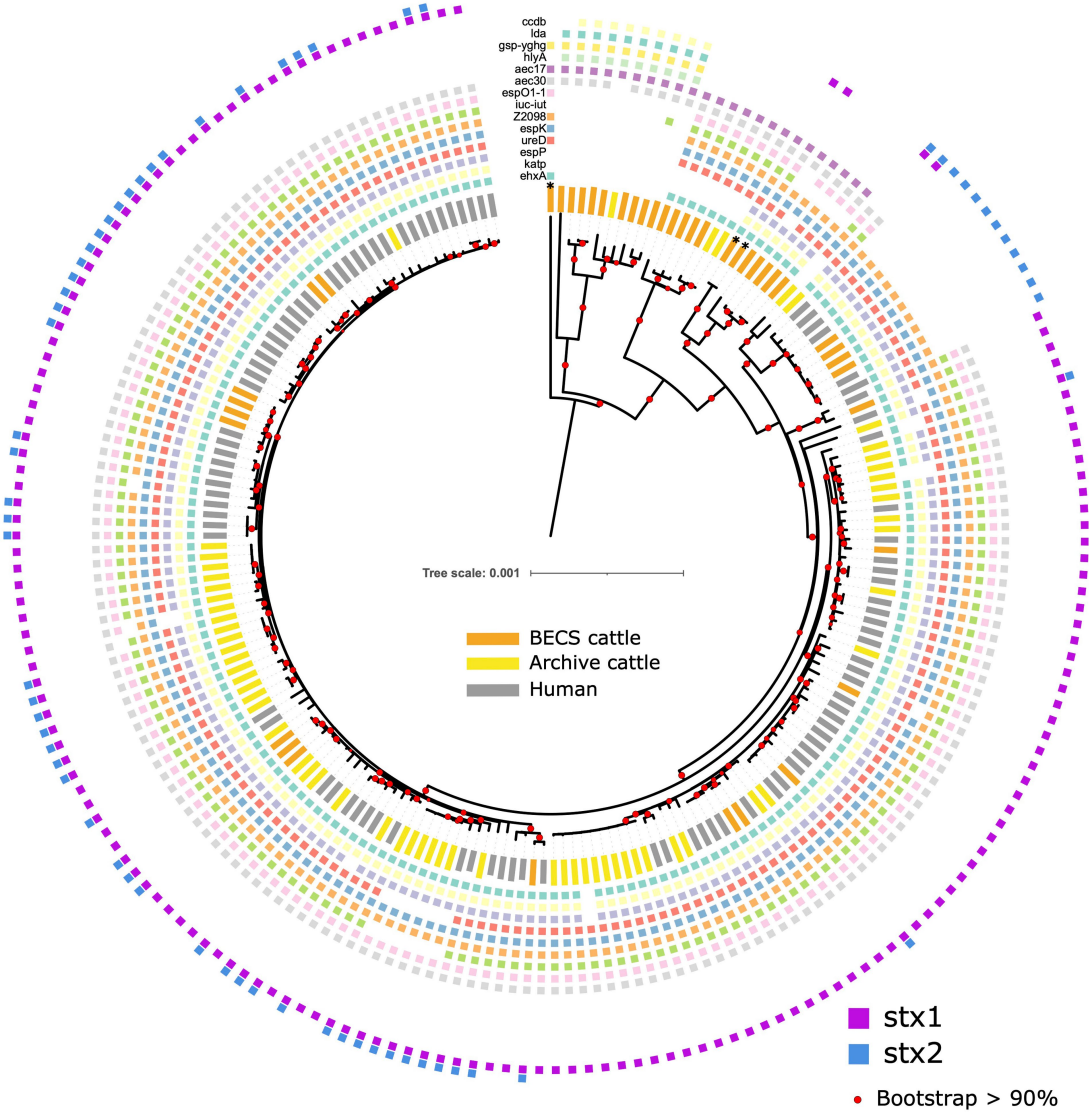
Maximum likelihood core-genome phylogenetic analysis of Scottish bovine and human O26:H11 strains (*n* = 195) and three O177:H11 strains (*), showing the presence of 14 selected key virulence genes or gene clusters. From inner to outer ring (*ehxA*, *katP*, *espP*, *ureD*, *espK*, *Z2098*, *iuc-iut*, *espO1-1*, *aec30*, *aec17*, *hlyA*, *gsp-yghg*, *lda*, and *ccdb*). Red circles indicate branches with >90% bootstrap support. Tree scale is in substitutions per site.

A total of 207 genes displayed differential occurrence, with distinct distributions noted according to *stx* status (Supplementary Table 7). All genomes were negative for the *etpD* gene, with 96.5% of all *stx*-positive strains (*n* = 167/173) showing the pVF profile typically observed in ST21 strains *ehxA*+/*katP*+/*espP*+/*etpD*- (Figure 5). Three *stx*-positive Archive bovine strains from different herds were *ehxA*-/*katP*-/*espP-*/*etpD*-, and one Archive bovine and one human strain were *ehxA*+/*katP*+/*espP-*/*etpD*-, with a further human strain having an *ehxA*+/*katP*-/*espP-*/*etpD*- pVF profile. The *stx*-negative ST396 strains bore an identical core virulence profile to the *stx*-positive strains, including the *ehxA*+/*katP*+/*espP*+/*etpD*- profile. A further 6 *stx*-negative Scottish cattle herds yielded 7 strains that were *ehxA*+/*katP*-/*espP*-/*etpD*-, whilst the remaining 9 *stx*-negative herds yielded 10 strains that were negative for all pVF genes. The *espL* and *fimB* genes showed similar distributions to *ehxA* across all *stx* profiles, excluding the *stx*-negative ST396 lineage, of which all but one strain was *fimB*-negative. All but one of the *stx*-negative strains, regardless of ST or pVF profile, also carried a distinct set of genes that were not detected in any *stx*-positive strains, including the genes *aec17, aec18*, *aec22*, *aec*23, *hcp,* and *vgrG,* which encode components of the type VI secretion system (Pukatzki et al., 2009), and the genes *Z0263* and *Z0265*.

#### 3.4.1. *stx*-positive strain virulence profiles

Other than *stx* subtype genes, the only gene specifically associated with *stx*-positive status was the iron regulatory protein 1 gene, *irp1*, detected in 98.9% (*n* = 171/173) of *stx*-positive strains, but not observed in *stx*-negative strains (Supplementary Table 7). In contrast, *irp2* was detected in all strains, regardless of *stx* status. This is unusual, given that *irp1* and *irp2* are typically found together within a high-pathogenicity island. However, further analysis of a subset of these genomes using the Artemis genome browser did identify an *irp1* variant allele in *stx*-negative strains, bearing a nine-base pair insertion sequence, which presumably reduced the alignment to below the set Abricate threshold parameters. The absence of *irp1* in the two *stx*-positive strains was found to be due to a contig break within the gene. A further gene associated with *stx*-positivity was *nleG5-1*. This gene was found in 97.7% (*n* = 169/173) of *stx*-positive strains, but was detected in only 9.1% (*n* = 2/22) of *stx*-negative strains.

Strains that were positive for *stx2*-only were distinguished from all other *stx* profiles, including negative strains, by the absence of the type VI secretory system gene *aec30*. However, other *aec* subtypes such as *aec17-19, 22, and 23* were either absent in all *stx* strains, but detected in the majority of *stx*-negatives, or in the case of subtypes *aec24-29*, detected across all categories. Additional genes that were absent in *stx2*-positive strains, but observed in all other *stx*-positive strains and in up to 50% of negative strains included the *espO1-1*, *iuc* and *iut* genes (Supplementary Table 7).

**Figure 6:**
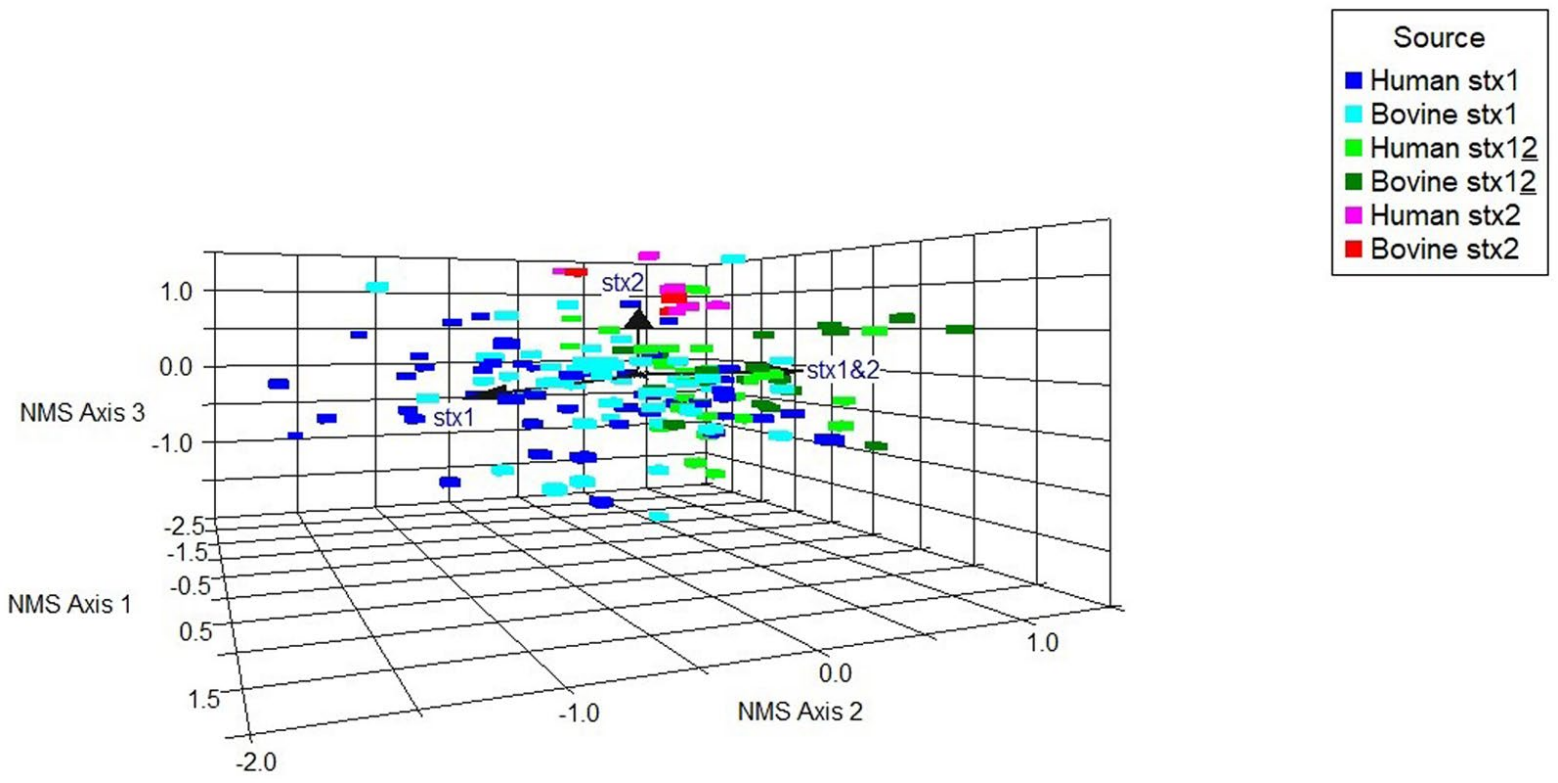
Non-metric multidimensional scaling ordination model to examine potential associations between a subset of differential gene occurrence for host source-*stx* profile combined (human, bovine; *stx1*: stx1, *stx2*: stx2, *stx1* + *stx2*: stx12), for all *stx*-positive genomes (*n* = 173). Arrows indicate vector direction.

A non-metric multidimensional scaling (NMS) ordination model was constructed to examine potential associations between binary virulence gene occurrence, *stx* profile, and host species for all *stx*-positive O26:H11 genomes (*n* = 173) (Figure 6; Supplementary Table 8A). A three-dimensional solution to the model was obtained, which explained 83.5% of the variation (axis 1 = 40.1%, axis 2 = 27.3%, and axis 3 = 16.1%). The graph was rotated to maximise the distance between cattle and human strains on axis 1 (Supplementary Figure 2). Axis 2 is explained by *stx1* (Kendall’s tau, −4.28) and *stx1* + *stx2* (Kendall’s tau, 0.465) strains. *Stx2* was located primarily on axis 3 (Kendall’s tau, 0.312) (Figure 6). A multi-response permutation procedure test (MRPP) found significant differences between human and bovine for *stx1* (bovine versus human, *p* = 0.002) and *stx1* + *stx2* (bovine versus human, *p* = 0.002). There were no differences for *stx2*-only strains between bovine and humans (MRPP, *p* = 0.158). Using Kendall’s tau as an indicator, most genes were not highly correlated with the NMS axes, though a weak to moderate association was observed for axis 3 and the non-LEE effector genes *nleC* and *nleG2-4* (Supplementary Table 8B). These data suggest that for our population, whilst some differences exist in virulence background between the differing *stx* subtypes and host source within the majority ST21 lineage, it was not possible to attribute this to specific genes. Virulence profiles within the *stx2a*-only clade were more conserved than for the *stx1* and *stx1* + *stx2* strains, which supports the phylogenetic analysis and observation that these strains have appeared in both Scottish cattle and human strain populations only relatively recently.

#### 3.4.2. *stx*-negative strain virulence profiles

The 22 s*tx*-negative strains from 19 herds were grouped into 2 core virulence profiles, “A” and “B,” across the ST29 and ST396 lineages, resulting in 3 distinct *stx*-negative populations overall (Figure 5; Supplementary Table 9).

The 12 “A” profile strains, which included all ST396 strains from four herds and ST29 strains from six herds, were located within the two clades phylogenetically closer to the *stx*-positive ST21 lineage. The majority of “A” strains carried *ehxA*, *Z2201,* and *espO1-1*, as well as the triplicate of genes *ureD*, *espK,* and *Z2098*, suggested as key markers for identifying *E. coli* with the potential for EHEC-type pathogenicity (Delannoy et al., 2013a). The presence of at least one of these three genes was always detected in all *stx*-positive bovine and human strains. ST396 is a less common ST variant of ST29, and all strains in this clade carried a greater complement of virulence genes, including the full *ehxA+*/*katP+*/*espP+*/*etpD*- pVF profile, as well as in all except one strain, the three *espK*, *ureD,* and *Z2098* genes. ST396 *stx2d*-positive O26:H11 strains bearing the *ehxA+*/*kapP+*/*espP+*/*etpD*- profile have previously been reported in a minority of human HUS cases from Italy (Michelacci et al., 2022).

The virulence gene profile borne by these “A” profile *stx*-negative strains, together with the phylogenetic grouping, is consistent with an ST29C1 clade classification (Ogura et al., 2017; Long et al., 2022) and suggests that the strains are EHEC-like derivatives. The loss and acquisition of *stx* genes from O26 strains, both *in vivo* and *in vitro*, has been previously documented (Bielaszewska et al., 2007; Senthakumaran et al., 2018). Current diagnostic reliance on PCR testing for *stx* and *eae* genes only, may therefore potentially result in false-negative classification of EHEC strains that have lost the *stx* gene during laboratory isolation or within host. Our data concur with the proposal by Delannoy et al. (2013a) that additional genes, including *ehxA*, *espK*, *ureD,* and *Z2098* should be included in diagnostic screening assays, and as shown here, are optimal gene markers for the identification of O26:H11 EHEC potential in livestock and animal products.

A further nine herds yielded 10 ST29 EPEC strains that were negative for *ehxA* and bore the virulence profile “B.” Distinguishing genes for this profile included *b2972*, the *gsp* cluster genes *C-M*, *yghg*, *hlyA*, *lda(A-I),* and *ccdb* (Figure 5; Supplementary Table 9). This virulence profile is consistent with the ST29C3 clade outlined by Long et al. (2022) and observed elsewhere (Leomil et al., 2005; Bugarel et al., 2011; Gonzalez-Escalona et al., 2016). Strains in this clade, whilst bearing a combination of virulence factors found across varying *E. coli* pathotypes (Kaper et al., 2004), do not appear to have the appropriate virulence background for EHEC pathogenicity following a potential recombination event with *stx*-bearing bacteriophage and are mostly represented by EPEC strains. The *b2972* locus (*pppA* gene), *yghG,* and *gsp(C-M)* are located in a common gene cluster associated with the Type II secretion system found in both pathogenic and non-pathogenic *E. coli* strains (Tauschek et al., 2002; Yang et al., 2007). The *pppA* and *yghG* genes are also associated with the regulation of heat-labile (LT) toxin (Strozen et al., 2012; Lu et al., 2016; Wang et al., 2020). *hlyA* encodes α-haemolysin, an important cytotoxin found in uropathogenic *E. coli* (Ristow and Welch, 2016), whilst *ccdb* encodes a cytotoxin present in the toxin-antitoxin system targeting *E. coli* gyrase and is carried by the F plasmid (Bernard and Couturier, 1992). The *lda* genes, present in the locus of diffuse adherence, encode adhesins associated with atypical EPEC and have also been reported in an O26:H11 paediatric clinical strain (Scaletsky et al., 2005).

One herd (BECS_2) yielded both ST21 *stx1a + stx2a*-positive and ST396 *stx*-negative strains bearing very similar key virulence genes, including *ehxA*+/*katP*+/*espP+*/*etpD*-, though missing *nleG-3*, *nleG2-4*, *nleG5-1,* and *fimB*. This herd also yielded an ST29 O177:H11 *stx*-negative strain, which, whilst *ehxA* positive, was *katP* and *espP* negative. In contrast, a second herd (BECS_27) yielded both *stx1a* and *stx*-negative O26:H11 strains bearing the “B” virulence profile. A third herd (BECS_18) generated an ST29 O26:H11 group “A” *stx*-negative strain and an O177:H11 *stx1a* strain, both of similar virulence profiles, though locating to different clades within the phylogeny.

### 3.5. Phage insertion site genes

**Figure 7:**
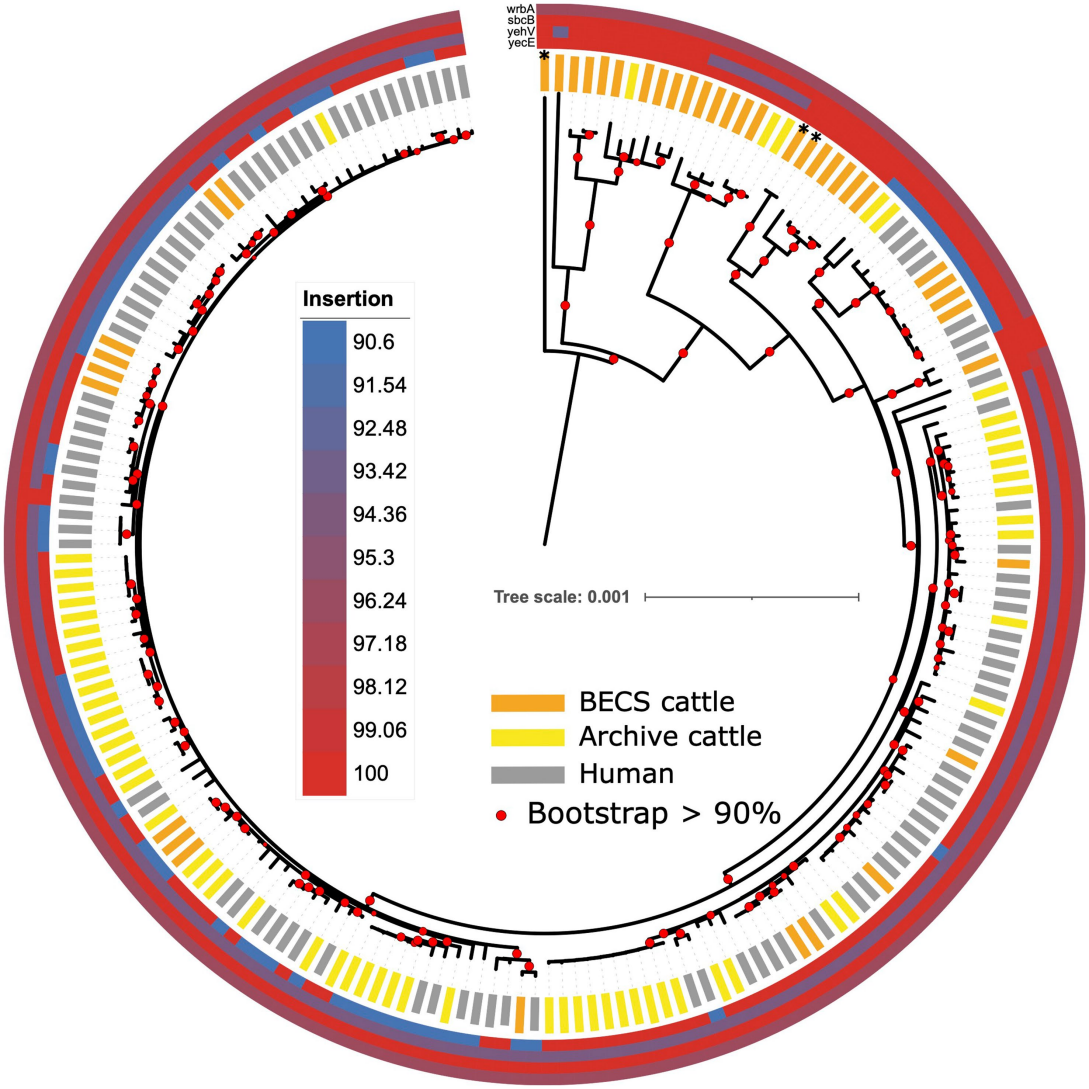
Maximum likelihood core-genome phylogenetic analysis of Scottish bovine and human O26:H11 strains (*n* = 195) and three O177:H11 strains (*) showing the sequence conservation of four known phage insertion sites (*yecE*, *yehV*, *sbcB*, and *wrbA*). Red circles indicate branches with >90% bootstrap support. Tree scale is in substitutions per site.

The *stx* gene is encoded by mobile bacteriophages, which integrates into the bacterial host genome at particular chromosomal insertion sites (Shaikh and Tarr, 2003; Rodríguez-Rubio et al., 2021). Insertion occurs within or adjacent to the host insertion site gene and typically causes disruption to the insertion site gene sequence. A number of integration sites have been identified for O26:H11 STEC, including the *yecE*, *wrbA*, *yehV,* and *sbcB* genes (Bonanno et al., 2015). In order to examine whether there was any evidence for the insertion of *stx*-phage at these sites across the collection of strains in this study, we compared the gene sequence identity obtained from the Abricate output with the presence and absence of *stx* and the *stx* subtype. We observed variability in nucleotide sequence percentage identity in the *stx* bacteriophage insertion site genes *yecE* and *yehV*, according to the *stx* status (Figure 7; Supplementary Table 10). *yehV* is one of the main insertion sites in O26:H11 for the *stx*1 bacteriophage and *yecE* for *stx2* (Bonanno et al., 2015). For *yecE*, variation in nucleotide percentage identity was significantly associated with the presence of the *stx2* gene (*p* < 0.001, test statistic 196, degrees of freedom 3), with 97% (64/66) of *stx2*-positive genomes showing a 90.6% identity and 98% (126/129) of *stx1*-only and *stx*-negative genomes showing 100% identity to *yecE*. In contrast, significant variation in percent identity for *yehV* appeared to be associated with *stx1* status (*p* < 0.001, test statistic 90.8, degrees of freedom 6), with the majority of the ST21 *stx1*-positive genomes, as well as a subset of *stx*-negative ST29, showing 94.13% identity to *yehV*. There was limited variability in percent identity across the genomes for *wrbA* and *sbcB*: 99% of genomes showed 96.37% identity to *wrbA* (193/195) and 100% identity to *sbcB* (194/195).

The individual insertion site gene size, sequence, and gene arrangements in the vicinity of the *yecE* and *yehV* genes were examined for a subset of bovine strains across the different *stx* categories using the Artemis genome viewer (Carver et al., 2012; Supplementary Table 1). In the *stx*-negative and *stx1*-positive genomes examined, *yecE* was 819 base pairs (bp) in size and was located within the consecutive gene sequences *yecD*, *yecE*, *yecN*, *cmoA,* and *cmoB*. In contrast, the four *stx2*-only positive bovine strains contained a truncated 111-bp fragment directly located next to the *yecD* gene, the latter immediately adjacent to a contig break. The truncated 111-bp fragment had 100% homology to bases 1–96 of the full-length *yecE.* A further 816 bp gene was located on an alternate contig at a different location within the genome, adjacent to the *yecN*, *cmoA,* and *cmoB* genes, in most cases flanked by an integrase gene. This 816 bp gene showed 0% homology to *yecE* between bases 1–70 and 99% homology between bases 71–819. All 13 *stx1* + *stx2* strains examined also showed a truncated 816 bp *yecE* gene adjacent to an integrase gene, and seven of these additionally bore the 111-bp fragment. This suggests the potential occurrence of an integration event at the *yecE* site, resulting in the disruption of this gene in the *stx2*-positive strains, which was not observed in the *stx1*-only and *stx*-negative strains.

We examined the *yehV* (*mlrA*), gene length, and arrangement in 6 *stx1*-positive, 13 *stx1* + *stx2,* and 22 *stx*-negative cattle O26:H11 genomes. All *stx1*-positive, 11/13 *stx1* + *stx2* and the 7 “A” profile ST29 *stx*-negative genomes showed <100% homology to *yehV* and contained a 648 bp gene of 94.13% identity to *yehV*, flanked in all cases by *yehW* and the integrase *IntQ_1* or *IntQ_2* genes (Hall et al., 2021). The presence of the truncated *yehV* gene flanked by integrase *intQ* genes is highly suggestive of a phage insertion event in these 7 “A” profile ST29 *stx-*negative strains. In contrast, 9/10 *stx*-negative ST29 profile “B” strains with 100% homology to *yehV*, as well as the three O177:H11 strains, contained a full-length 732 bp *yehV* (*mlrA*) gene, flanked by *yehW* and the sensory histidine kinase gene *ypdA-1*. One “B” profile ST29 *stx-*negative strain contained two smaller gene fragments, and two *stx1* + *stx2* strains bore the full-length gene.

The ST396 “A” profile *stx*-negative strains carried a greater complement of virulence genes than the ST29 *stx-*negative strains. However, the *yehV* local gene arrangement in these ST396 strains did not show evidence of phage integration or interruption, with an intact 732 bp *yehV* gene. We examined the gene arrangement in these strains at another potential bacteriophage insertion site, the *torS-T* intergenic region (González-Escalona et al., 2019); however, this region was uninterrupted in all the ST396 strains.

All *stx*-negative cattle genomes were submitted to PHASTER for the identification of any *stx*-prophage regions; however, the results were inconclusive (Supplementary Table 9). Both intact and incomplete prophage regions with homology to *stx*-prophage as the first or second-listed most common phage were identified in the majority of the *stx*-negative strains. Due to the limitations of short-read genome assembly, long-read sequencing would be required to confirm the presence of any inserted prophage in *stx*-negative strains. However, given the distinct gene arrangement in the locality of *yehV,* together with the virulence background present in the “A” profile strains, it would seem probable that they had either the potential for acquiring *stx* or had previously been *stx*-positive and subsequently lost the *stx* gene from an integrated prophage.

### 3.6. Antimicrobial resistance profiles of bovine and human strains

Acquired antimicrobial resistance genes (ARGs) were identified *in silico* for 12.1% (12/99) of the O26:H11 bovine strains, 15.6% of the cattle herds (10/64) (Table 2). The commonest genes detected were *sul2* and *tet*, found in six herds; *aph(3″)* and *aph(6″)* in five herds; and *bla*_TEM_ and *dfrA*, each detected in four herds. Three-quarters of resistant strains (9/12) were positive for more than one ARG (median 4.5, range 1–5), with the commonest combination being *aph(3″)*, *aph(6″)*, together with one or more further ARGs. Seventeen human strains (17.7%) carried ARGs (Table 2), with the commonest being *aph(3″)* and *aph(6″)* in 13 strains, followed by *sul2* and *floR* in eight strains. Multiple resistances were also common in human strains (median 4, range 1–9).

**Table 2:** Summary of antimicrobial resistance genes detected in the Scottish bovine and human O26:H11 genomes by STARAMR and ResFinder databases, where P indicates genome positive for respective genes.

Strain ID	Source	*ant(3″)* or *aadA1*	*aph(3″)-Ib*	*aph(6″)-Id*	*bla*_OXA-1_	*bla*_TEM-1B_	*bla*_TEM-1C_	*bla*_TEM-30_	*dfrA1*	*dfrA5*	*floR*	*mph(B)*	*sul1*	*sul2*	*tet A*	*tet B*	*tet C*
XH2001256	Archive_31															P	
XH2001264r	Archive_14	P							P		P		P	P			
XH2001404	Archive_35		P	P				P						P	P		
XH800939X	BECS_2		P	P			P		P					P			
XH800941P	BECS_2		P	P			P		P					P			
XH800951Y	BECS_16		P	P			P		P					P			
XH800956T	BECS_25									P					P		
XH800958M	BECS_27		P	P		P								P	P		
XH800985H	BECS_12		P	P							P			P			
XH800986Y	BECS_12		P	P							P			P			
XH800989A	BECS_24																P
XH801004W	BECS_6																P
SME-18-85	Human					P											
SME-18-152	Human		P	P		P								P			
SME-18-190	Human	P	P	P					P		P	P	P	P	P		
SME-18-45	Human		P	P													
SME-18-27	Human		P	P		P					P			P	P		
SME-18-201	Human		P	P		P					P			P	P		
SME-18-138	Human	P											P		P		
SME-18-30	Human		P	P							P			P			
SME-18-194	Human		P	P			P							P			
SME-18-88	Human		P	P							P			P			
SME-18-10	Human		P	P				P									
MUOON6	Human		P	P													
SME-19-228	Human		P	P							P						
SME-18-195	Human		P	P													
SME-19-812	Human													P			
SME-20-404	Human	P			P						P			P			
SME-20-481	Human		P	P							P			P			

Strains carrying resistance were screened for the presence of mobile genetic elements (Supplementary Table 11). One bovine Archive genome carried a clinical class 1 integron with a typical cassette arrangement of 5′-*intI1*, *dfrA1*, *aadA1*, *qacEΔ1*, and *sul1*-3′ with an additional two genes, *floR* and *sul2,* found in close proximity on the same contig. Two multiply-resistant human strains also carried clinical class 1 integrons. Across all genomes, *bla*_TEM_ genes were found on transposon Tn2 in six cases, and in cattle, *dfrA1* was associated with the composite transposon cn_4568_IS26 in two herds.

The resistome seen in the bovine isolates was very similar to that observed in the Scottish human dataset, with the commonest ARGs conferring resistance to streptomycin and spectinomycin aminoglycosides, sulphonamides, tetracyclines, and beta-lactam agents such as ampicillin, the latter class designated as critically important antimicrobials for human health by the World Health Organization (2019). The proportion of the bovine strains carrying ARGs is in line with that reported in a collection of O26:H11 strains from home (non-travel-associated) human STEC O26 cases from England and Wales isolated during 2015 (Day et al., 2017), but slightly lower than described in a more recent report on human case clonal complex 29 STEC isolates in England between 2014 and 2021 (Rodwell et al., 2023). Our results differ markedly from the very high AMR prevalence reported in a collection of O26:H11 strains from feedlot cattle in the United States (Gonzalez-Escalona et al., 2016); however, this likely reflects differences in the management systems and associated antimicrobial usage of the livestock systems between the two countries.

Overall, these data are in accordance with current antimicrobial usage observed within the bovine sector in the UK, with beta-lactams, tetracycline, and streptomycin being the most frequently prescribed antimicrobials in both beef and dairy cattle (Humphry et al., 2021; RUMA, 2021). Given ruminants are the primary reservoir source for human infection within the UK and antimicrobial therapy is generally not indicated in human STEC infection (Tarr and Freedman, 2022), this agreement in the resistance profile between the Scottish cattle and human genomes is not unexpected.

## 4. Conclusion

In conclusion, within the study herds, all *stx*-positive cattle O26:H11 strains fell within the ST21 lineage, and no ST29 *stx-*positive strains were identified. Bovine and clinical human strain genomes were relatively well interspersed, with the *stx* subtype generally clade-specific. Highly pathogenic *stx2a*-only ST21 was identified in two herds from the second cattle survey and in human strains from 2010 onwards. Where multiple strains were available from individual herds, we observed limited variability within the *stx* subtype, suggesting that the same *stx* subtype strains typically spread clonally at the farm level rather than supporting multiple lineage introductions across a cohort. Half of the *stx*-negative survey herds yielded O26:H11 strains with virulence profiles similar to those observed in *stx*-positive strains, including the genes *ehxA*, *espK*, and *Z2098*, which have been proposed as markers for “EHEC-like” potential. These data suggest that the reservoir of O26:H11 in Scottish cattle bearing a genomic background compatible with EHEC potential and therefore of public health concern may be greater than would be expected based on the detection of the STEC markers *stx* and *eae* alone.

## Data availability statement

The datasets presented in this study can be found in online repositories. The names of the repository/repositories and accession number(s) can be found at: https://www.ebi.ac.uk/ena, PRJEB57355; https://www.ncbi.nlm.nih.gov/, PRJNA419720.

## Ethics statement

The studies involving humans were approved by the biorepository bank for the National Health Service (NHS) Lothian, Scotland, covering the sequencing of samples and use of de-identified bacterial genomes from human clinical samples (20/ES/0061). The studies were conducted in accordance with the local legislation and institutional requirements. The human samples used in this study were acquired from a by- product of routine care or industry. Written informed consent for participation was not required from the participants or the participants’ legal guardians/next of kin in accordance with the national legislation and institutional requirements. Ethical approval was not required for the studies involving animals in accordance with the local legislation and institutional requirements, because this study uses only strains from cattle faecal pat samples obtained from the BECS study doi: 10.1017/S0950268817002151 and from the national survey reported in doi: 10.1128/AEM.72.1.653-659.2006 conducted by Scotland’s Rural College (SRUC). Scotland’s Rural College did not require the study to be reviewed or approved by an ethics committee as per the local legislation where the studies were conducted, because samples were industry by-products (cattle faecal pats) collected from the ground of grazing land and the floor of pens, which involved no animal contact or intervention. The funding bodies approved and authorised informed consent documentation for farm survey participants, to enable collection of dropped faecal pat samples from participant land. Permission had been granted and written consent obtained from farm participants for the samples, strains and data to be used for further research. All farm participant personal data were handled in accordance with the UK Data Protection Act (1998) and are now handled in accordance with the UK General Data Protection Regulation (GDPR 2018).

## Author contributions

DH: Conceptualization, Data curation, Formal analysis, Funding acquisition, Investigation, Project administration, Writing – original draft, Writing – review & editing. BW: Data curation, Formal analysis, Visualization, Writing – original draft, Writing – review & editing, Investigation. KM: Investigation, Writing – review & editing. MC-T: Formal analysis, Writing – review & editing. AB: Investigation, Writing – review & editing. ST: Resources, Writing – review & editing. DG: Conceptualization, Writing – review & editing. SD: Writing – review & editing. PF: Writing – review & editing. MP: Writing – review & editing. GG: Resources, Writing – review & editing. AH: Conceptualization, Data curation, Investigation, Resources, Writing – original draft, Writing – review & editing. LA: Conceptualization, Data curation, Investigation, Resources, Writing – original draft, Writing – review & editing.

## Footnotes

^1^ https://enterobase.warwick.ac.uk/schemes/Escherichia.cgMLSTv1

^2^ https://github.com/phac-nml/ecoli_vf

^3^ https://cge.food.dtu.dk/services/MobileElementFinder/

^4^ https://blast.ncbi.nlm.nih.gov/Blast.cgi

^5^ https://www.uniprot.org/

^6^ https://phaster.ca/
