# Supplementary material for: Phylogenetic relationship and virulence composition of Escherichia coli O26:H11 cattle and human strain collections in Scotland; 2002–2020
Source: Front Microbiol. 2023 Nov 6;14:1260422. doi: 10.3389/fmicb.2023.1260422 (PMC10657854; doi:10.3389/fmicb.2023.1260422)

SUPPLEMENTARY FIGURES

Supplementary Figure 1. Correlation matrix showing genes that were removed from the nonmetric multidimensional scaling analysis due to high pairwise correlation between the genes. Strength of correlation is represented by colour, where blue denotes high correlation and brown denotes low correlation between gene pairs.

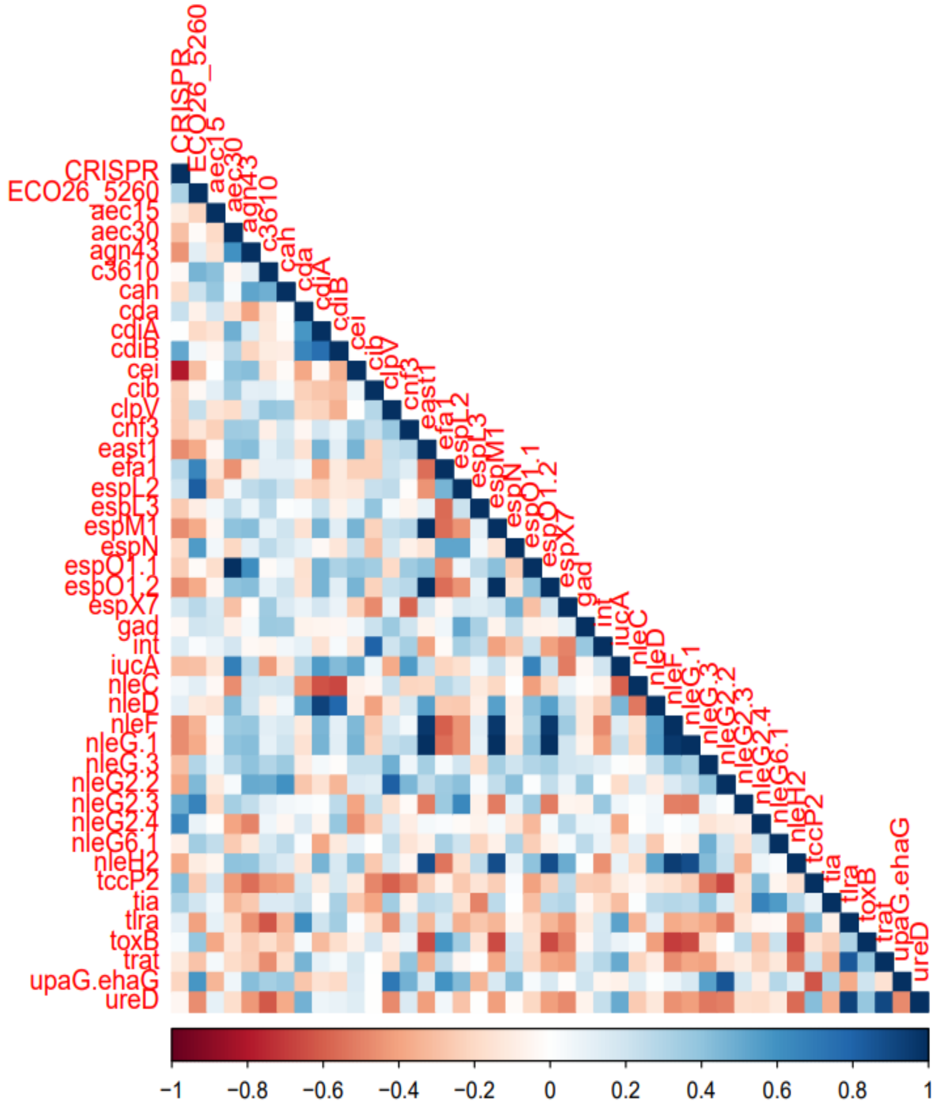

Supplementary Figure 2. A non-metric multidimensional scaling ordination model was constructed to examine potential associations between a subset of differential gene occurrence according to host source (bovine, human). Note Abricate analysis for gene detection used threshold parameters of minimum % identity 80 and minimum % coverage 80.

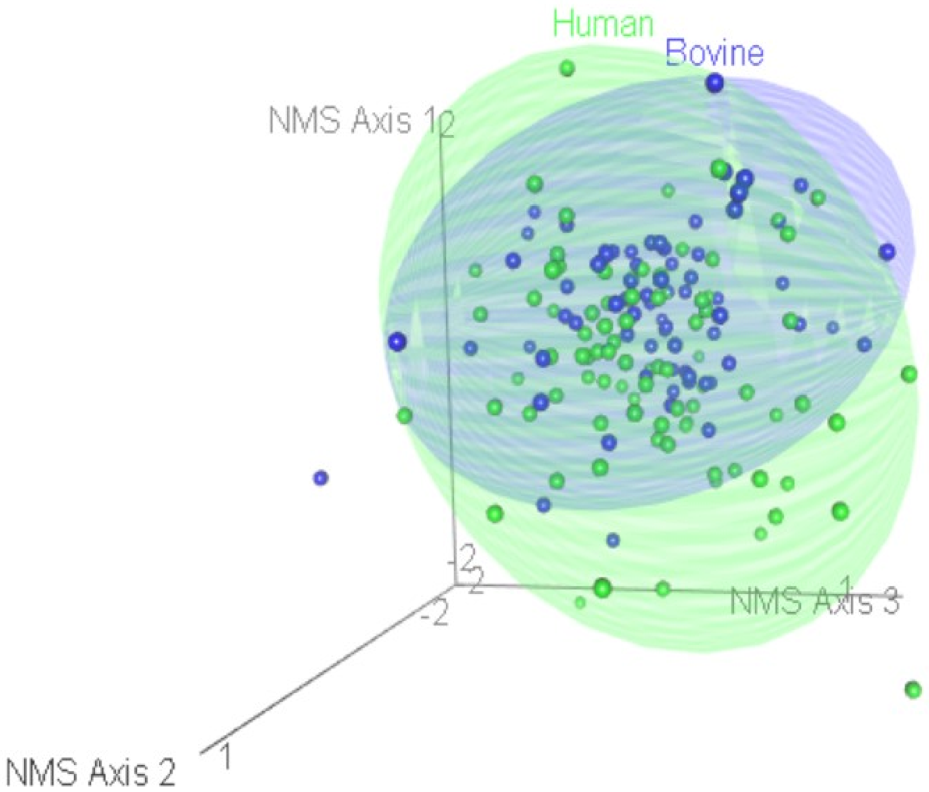

Supplement: Supplementary file 1 [file Data_Sheet_1.ZIP › Supplementary Figure 1 and Figure 2_210923.pdf]
