# Supplementary material for: Phylogenetic relationship and virulence composition of Escherichia coli O26:H11 cattle and human strain collections in Scotland; 2002–2020
Source: Front Microbiol. 2023 Nov 6;14:1260422. doi: 10.3389/fmicb.2023.1260422 (PMC10657854; doi:10.3389/fmicb.2023.1260422)
Supplement: Supplementary file 1 [file Data_Sheet_1.ZIP › Supplementary Table 5 and Table 6_210923.pdf]

Supplementary Table 5: Summary statistics for pairwise SNP differences comparisons between genomes, for Scottish cattle and human strain genomes. Only genome pairs that were within 200 pairwise SNP differences were included in the analysis, showing the pairwise SNP minimum (Min), median, maximum (Max), quartile (Q) and interquartile range (IQR).

**Comparison categories**	**Number of genomes within 200 SNP**	**Number of pair matches**	**Pairwise SNP difference**					
			**Min**	**Q 1**	**Median**	**Q 3**	**Max**	**IQR**
All Cattle to Cattle	99	2409	0	61	108	126	199	65
Within herds, Combined surveys	54	86	0	1	3	8	128	7
Within herds, Same survey	52	79	0	1	3	6	128	5
Within herds, Between surveys	7	7	69	69	69	69	126	0
Between herds, Combined surveys	97	2323	6	63	110	126	199	63
Between herds, Same survey	94	1502	6	60	110	123	199	63
Between herds, Between surveys	85	821	20	69	110	131	199	62
Between regions, Combined surveys	97	1981	10	64	112	127	199	63
Between regions, Same survey	94	1270	10	62	112	124	198	62
Between regions, Between surveys	84	711	20	69	112	132	199	63
Within Region, Combined surveys	89	428	0	28	67	115	199	87
Within region, Same survey	84	311	0	12	44	112	199	100
Within region, Between surveys	63	117	22	69	78	125	157	56
Within region, Different herd, Combined surveys	77	342	6	44	76	121	199	77
Within region, Different herd, Same survey	70	232	6	42	67	117	199	75
Within region, Different herd, Between surveys	59	110	22	71	86	126	157	55
Scottish Cattle to Scottish Human	169	6097	9	69	114	135	178	66
Scottish Human to Scottish Human	94	3843	0	74	118	144	180	70

Supplementary Table 6: Summary statistics for pairwise SNP difference comparisons between Scottish cattle strain genomes and external country O26:H11 genomes, downloaded from EnteroBase (*E.coli* ST29 complex). Only external country genomes that were within 200 pairwise SNP difference to Scottish cattle genomes were included; showing the pairwise SNP minimum (Min), median, maximum (Max), quartile (Q) and interquartile range (IGR).

Country	Total country genomes	Number of genomes within 200 SNP	Percent of country genomes within 200 SNPs	Number of pair matches	Pairwise SNP difference					
					Min	Q1	Median	Q3	Max	IQR
Argentina	2	0	0.0	0	-	-	-	-	-	-
Australia	92	14	15.2	236	82	120	191	196	199	76
Austria	7	4	57.1	240	175	186	191	195	199	9
Belgium	92	71	77.2	1500	44	103	122	145	199	42
Canada	81	26	32.1	171	10	72	136	155	187	83
Chile	2	2	100.0	70	157	168	173	177	198	9
China	11	0	0.0	0	-	-	-	-	-	-
Czechia	6	5	83.3	328	61	82	125	140	199	58
Denmark	38	27	71.1	1046	69	172	182	188	199	16
Ecudaor	4	0	0.0	0	-	-	-	-	-	-
Finland	2	2	100.0	47	93	189	192	197	199	8
France	338	262	77.5	3256	21	102	124	144	199	42
Gambia	4	0	0.0	0	-	-	-	-	-	-
Germany	64	38	59.4	1629	59	152	185	192	199	40
Guinea-Bissau	2	0	0.0	0	-	-	-	-	-	-
Italy	20	12	60.0	49	31	92	102	166	199	75
Japan	219	180	82.2	1211	74	171	190	195	199	24
Kenya	2	0	0.0	0	-	-	-	-	-	-
Lebanon	1	0	0.0	0	-	-	-	-	-	-
Luxembourg	4	3	75.0	9	79	83	99	106	144	23
New Zealand	34	7	20.6	12	109	120	148	149	152	30
Norway	1	1	100.0	69	163	174	179	183	199	9
Poland	2	1	50.0	1	125	*	125	*	125	*
Portugal	3	2	66.7	5	72	73	78	139	199	67
Romania	1	0	0.0	0	-	-	-	-	-	-
South Africa	7	6	85.7	114	63	82	131	191	199	109
South Korea	3	2	66.7	7	193	194	198	199	199	5
Spain	6	1	16.7	69	30	53	116	126	154	73
Sweden	3	1	33.3	4	102	102	106	109	109	6.5
Switzerland	4	2	50.0	70	58	73	83	135	153	62
Tanzania	1	0	0.0	0	-	-	-	-	-	-
Thailand	2	0	0.0	0	-	-	-	-	-	-
UK exc Scot	1217	883	72.6	25569	7	86	123	145	199	59

US	6054	2417	39.9	8286	26	135	172	190	199	55
Uruguay	1	0	0.0	0	-	-	-	-	-	-
Vietnam	2	0	0.0	0	-	-	-	-	-	-
